# Conceptualizing and implementing a health workforce registry in Nigeria

**DOI:** 10.1186/s12960-022-00706-3

**Published:** 2022-01-15

**Authors:** Sunny C. Okoroafor, Agbonkhese I. Oaiya, David Oviaesu, Adam Ahmat, Martin Osubor, Jennifer Nyoni

**Affiliations:** 1 World Health Organization Country Office in Nigeria, United Nations House, Plot 617, Diplomatic Zone, Central Area District, Abuja, Nigeria; 2 Independent Consultant, Abuja, Nigeria; 3 World Health Organization Regional Office for Africa, Brazzaville, Congo; 4 Global Affairs Canada, Abuja, Nigeria

**Keywords:** Health workforce registry, Nigeria Health Workforce Registry, HRH information system, HRH evidence

## Abstract

**Background:**

Nigeria’s health sector aims to ensure that the right number of health workers that are qualified, skilled, and distributed equitably, are available for quality health service provision at all levels. Achieving this requires accurate and timely health workforce information. This informed the development of the Nigeria Health Workforce Registry (NHWR) based on the global, regional, and national strategies for strengthening the HRH towards achieving universal health coverage. This case study describes the process of conceptualizing and establishing the NHWR, and discusses the strategies for developing sustainable and scalable health workforce registries.

**Case presentation:**

In designing the NHWR, a review of existing national HRH policies and guidelines, as well as reports of previous endeavors was done to learn what had been done previously and obtain the views of stakeholders on how to develop a scalable and sustainable registry. The findings indicated the need to review the architecture of the registry to align with other health information systems, develop a standardized data set and guidance documents for the registry including a standard operating procedure to ensure that a holistic process is adopted in data collection, management and use nationally. Learning from the findings, a conceptual framework was developed, a registry managed centrally by the Federal Ministry of Health was developed and decentralized, a standardized tool based on a national minimum data was developed and adopted nationally, a registry prototype was developed using iHRIS Manage and the registry governance functions were integrated into the health information system governance structures. To sustain the functionality of the NHWR, the handbook of the NHWR that comprised of an implementation guide, the standard operating procedure, and the basic user training manual was developed and the capacity of government staff was built on the operations of the registry.

**Conclusion:**

In establishing a functional and sustainable registry, learning from experiences is essential in shaping acceptable, sustainable, and scalable approaches. Instituting governance structures that include and involve policymakers, health managers and users is of great importance in the design, planning, implementation, and decentralization stages. In addition, developing standardized tools based on the health system's needs and instituting supportable mechanisms for data flow and use for policy, planning, development, and management is essential.

## Background

The Global Strategy for Human Resources for Health: Workforce 2030 envisions attaining universal health coverage (UHC) and the Sustainable Development Goals (SDG) by ensuring equitable access to health workers [1]. This is informed by evidence that the level of functionality of health systems in the development and humanitarian contexts depends on the availability, accessibility, acceptability, and competencies of health workers [1–3]. Key health workforce challenges impeding the achievement of health sector goals include poor health workforce planning, the inequitable distribution of health workers by geographical location and levels of care, and imbalances in the production of health workers based on needs and skill-mix for health service delivery [4]. Addressing these challenges requires adequate and up to date health workforce information that is contemporary and comprehensive.

Many countries in Africa face challenges of collecting, analyzing, publishing and using health workforce information for policy development and planning [5]. An assessment of the human resources for health (HRH) strategic plans in the Africa Region showed that 92% of country HRH plans earmarked strategies to improve the availability of health workforce information by improving their human resource information system (HRIS) (including health workforce registries) and using the health workforce information to inform HRH planning and management in the public and private sector [5]. In addition, 17% of the countries identified the need to build the capacity of health and HRH managers in data generation, analysis, and use, as well as research and monitoring, and evaluation [5].

The establishment of health workforce registries with minimum data set is recommended by the World Health Organization for strengthening health systems in countries [1]. The establishment of registries in countries has been reported to facilitate improvement in the distribution and deployment of health workers [6]. Studies have also reported that the introduction and use of information from health workforce registries contribute to improving the skill-mix of health workers, informed scale-up of health workforce development, and improved staffing and availability of quality HRH information for health and HRH planning [4, 6–12].

Over the years, access to quality HRH information has been a challenge in Nigeria. Where they exist, they are stored in paper formats or multiple non-interoperable databases and under the control of several entities that often do not communicate with each other [13]. Thus, aggregation and analyses for evidence-based planning have been a prolonged challenge. This was exacerbated by weak HRH governance and coordination for HRH data and its effect on access to information for planning and management of the health workforce [14–16].

Nigeria’s health sector and HRH strategic plans aim to ensure that the right number of health workers that are qualified, skilled, and distributed equitably are available for quality health service provision at all levels [17, 18]. Achieving this requires accurate and timely health workforce information. This informed the development of the Nigeria Health Workforce Registry (NHWR) based on the global, regional, and national strategies for strengthening the HRH towards achieving the UHC and the SDG [17, 18], and the benefits of health workforce registries [4, 6–12]. The World Health Organization with funding from the Global Affairs Canada supported the Federal Ministry of Health (FMOH) to achieve this. This case study describes the process of conceptualizing and establishing the NHWR, and discusses the strategies for developing sustainable and scalable health workforce registries.

## Case presentation

### Context

Previously, various partners have been supporting national and sub-national entities to develop health workforce registries. This resulted in multiple non-interoperable systems at various levels that were not integrated at the federal level to ease collation and planning as well as duplicity in costs of hosting and maintaining the systems [19]. Over time, most of these multiple systems ceased to exist.

In 2015, the NHWR Operational Guidelines was developed, and approved by the 57th session of the National Council on Health. The operational guideline was developed based on the World Health Organization (WHO) guidance on minimum data set for health workforce registries [20] with the recommended data set adapted to the context. Beyond the data set, the operational guidelines also provide guidance on the implementation arrangement, governance structure, and security mechanism for the NHWR.

### Conceptualization

In designing the NHWR, a review of existing national HRH policies and guidelines, as well as reports of previous endeavors was commissioned by the FMOH in 2018. A NHWR operational team that comprised of staff of HRH unit, ICT department and partners conducted this review. This team held several consultations with representatives from Governments’ ministries, departments, and agencies at national and sub-national levels, and some development partners. The essence was to learn what had been done, what existed, strategies employed previously and obtain the views of stakeholders on how to develop a scalable and sustainable registry.

The key findings included the existence of some proprietary systems developed with the support of partners and the 2015 National Operational Guidelines for the NHWR [21]. The existing systems were not developed using a nationally validated and standardized tool, and did not capture information identified in the operational guidelines. The existing registries also did not have up-to-date information on the stock and distribution of health workers, mainly because there were no guidelines for updating and orienting users on the systems, no standard operating procedure to guide data flow and management, the high attrition rate of trained government stakeholders, and no budget lines for collection, collation and importing of health workforce information.

Synthesis of the findings highlighted the need to review the architecture of the registry to align with those of other health information systems in the country, such as the District Health Information system (DHIS). It also indicated the need to develop a standardized data collection tool and registry prototype based on the agreed and approved data set for the registry, and guidance documents to guide the programmatic process of decentralizing the registry. A guide for health workforce data management, updating, and reporting on the registry was also needed to ensure that a holistic process is adopted nationally.

### Review of the registry architecture

To streamline the multiple systems developed at various levels with no access centrally at the FMOH level, a review of architecture was needed. To develop a sustainable NHWR, we revised the architecture to align with the already functional DHIS. Thus, only one registry managed centrally by the FMoH was developed. This NHWR was designed to have information for all the submitting entities with approved focal points having access to manage the health workforce information relevant to them. We aligned the revised architecture with the policy thrust of having a web-enabled system to serve as a national repository for accurate and timely information on all health workers under the employment of national and sub-national entities and link them to administrative units, facilities, etc. [13, 21]. This new architecture was approved by the government for roll-out as it was projected to improve efficiency and effectiveness in the functionality of the registry, substantially reduce the personnel and hosting costs for multiple systems, and streamline backup and software maintenance operations for the registry.

### Tool development

The findings of the review indicated the importance of developing a standardized tool for the registry. The minimum data set outlined in the NHWR operational guidelines [21] was used to inform the development of a tool. The tool comprised of the 11 data sets that included the specified 10 minimum data set and an additional data set that captured educational history [13, 21]. The additional data set was included by government stakeholders to capture information on health workers who belong to several professionally regulated cadres and those not regulated and licensed for practice, such as field epidemiologists, health leadership certifications/qualifications, and post-graduate trainees. The minimum data set was identification number, full name, birth history, citizenship, country of residence and language, address, contact information, professional license and certification, employment status, employment address, and data submission institution. The minimum data set comprised 65 elements capturing details on demographic details of institutions and health workers, unique national and professional identifiers, detailed professional and educational details, and contact details of institutions and health workers. Based on the nationally recognized and professionally regulated cadres, 51 categorizations of health workers, including specialties in the country are also included to guide the development of country profiles [13]. These data sets and elements were used to develop a paper-based questionnaire and MS-excel based data collation tool [13]. These tools were piloted in two states (Bauchi and Cross River States) and validated by health and HRH planning managers, and partners.

### Registry design

Technical assistance was provided to government stakeholders to conduct a software analysis to identify the software technology and database for the registry as well as a web-hosting facility. Key considerations that guided the process were propriety, ease of use, interoperability with other health information systems, use of the system in other countries, and availability of technical expertise within the region for assistance and ease of adaptability. iHRIS Manage, a free and open-source web-based human resource application was selected to serve because of several benefits that include no software licensing, ease of customization, auditability, and interoperability with other existing health information systems, such as DHIS2 amongst others [13].

Subsequently, the prototype of the registry with agreed data sets and elements was developed and the system was hosted in an FMoH approved web-hosting facility.

### Governance structures

The national operational guidelines [21] provided for the establishment of a national steering committee for the registry which will serve as the highest decision-making and coordination group for the implementation and operations of the registry. The guideline also indicated that this body would be replicated at the state level.

In 2019, the leadership of the FMoH integrated the NHWR governance functions to the health information system governance structures [16, 22]—the health data governance council (HDGC) and the health data consultative committee (HDCC). This was done to ensure integration of the HRH information into the national health information system, leverage the functional health information system governance mechanisms, and avoid duplication of governance structures for health information systems. This approach also promoted sustainability as the HDGC and HDDC have been functional for several years in the country and their functions have been decentralized to sub-national levels [16, 22].

The HDGC provides leadership and direction on health information system investments and data use in Nigeria. This body is available at the national and states with the Minister of Health and Commissioners of Health chairing at national and sub-national levels, respectively. The Health Data Consultative Committee (HDDC), comprising the technical staff of government and partners involved with the routine management of the health information system, provides evidence and supports the HDGC [16, 22].

The function of operationalizing the NHWR was delegated to the NHWR operational team that comprises the technical staff of the HRH and ICT department teams. This team leads the decentralization of the registry and provides technical assistance to the HRH focal persons that manage the registry at the submitting entities.

### Rollout activities

Following the finalization of the tools, development of the registry prototype and orientation by health sector leadership on the governance mechanisms, roll out of the registry was piloted in Bauchi and Cross River States in 2018. Following the pilot, the decentralization to nine other states ensued in 2019 and 2020 with funding obtained from various sources [23]. The States are Abia, Adamawa, Anambra, Borno, Edo, Niger, Osun, Sokoto and Yobe [23]. Overall, the NHWR was decentralized to 11 of the 36 states in Nigeria that were purposefully selected. These 11 states have an estimated population of 56 million representing ~ 28% of Nigeria’s population.

To obtain political commitment and buy-in from the stakeholders, we engaged and oriented state-level authorities from the Ministry of Health, its agencies, and parastatals, Office of the Head of Service, Civil Service Commission, and Local Government stakeholders. This commitment served as a milestone for initiating activities to roll out the registry and their leadership in coordinating and sustaining activities during this process. They also provided contextual guidance that informed the development of state-specific strategies and plans for developing the nodes of the registry.

Aligning with the guidance provided at the federal level, state level HDGC and HDCC were engaged through an operational team comprising of the Directors of Health Planning of the Ministry of Health and Primary Health Care Development Agency, HRH focal persons, and primary health care coordinators. These groups were also oriented on the process and the commencement of the rollout was approved by the Commissioners for Health.

The operational teams were trained on the implementation process and standard operating procedure of the registry in a workshop. During the workshop, the state implementation strategies and plans were finalized and a microplanning exercise was conducted. This exercise served to map existing health workers in the states by the ministry, department, agency, parastatal, local government area, ward, and service delivery point. The microplans were used to plan the data collection process, the printing and distribution of the paper-based data collection tools, and data management processes and timelines.

### Data collection and management

The operational team led the deployment of the data collection forms in the health sector departments, agencies, and parastatals, as well as at local government, ward, and service delivery levels. They also obtained the soft copies of the previous month’s nominal rolls which were used to triangulate the information obtained during the primary data collection process. The paper-based tool was completed by all health workers in the States and returned through government channels back to the operational team. The operational team reviewed each completed form to ensure completeness and confirmed that incomplete forms were thoroughly completed by the health worker. This process that lasted for an average of 6 months was supervised by the staff of WHO and senior members of the HDDC. Completed data collection forms for all health workers in the health sector were collated by relevant submitting entities and submitted to the Director of Health Planning in the State’s Ministry of Health.

Following data collection, a residential workshop was conducted for the data entry, cleaning, standardization, storage, and analysis in each state. This was supervised by HDDC. During this workshop, health workforce information was entered into the data collation tool by data entry clerks, standardized, and collated by the submitting entity. Preliminary analysis of the information was done by submitting entity with a draft public health sector profile for the States developed.

Validation workshops were conducted in each State to validate the health workforce information with all health, personnel, and HRH managers from the submitting entities and PHC coordinators in attendance. During these meetings, the total HRH stock in the state by ward, and submitting entity presented in the data collation template and draft public health sector profile were validated.

The validated information was imported into the NHWR under the supervision of the HDDC and operational team and basic user training on the NHWR was conducted for the HDDC and operational team members. The training covered the implementation process, standard operating procedure, and operations of the registry using the demo and live Registry systems. Subsequently, other capacity-building actions were undertaken on data processing and use. They included training, mentoring, development of how-to videos and guides.

### Sustaining the registry

To sustain the functionality of the NHWR, following the pilot of the registry implementation in Bauchi and Cross River States, the FMoH was supported to finalize the handbook of the NHWR that comprises of an implementation guide, the standard operating procedure, and the basic user training manual. In addition, an advanced user manual and a disaster recovery plan were developed.

**Fig. 1:**
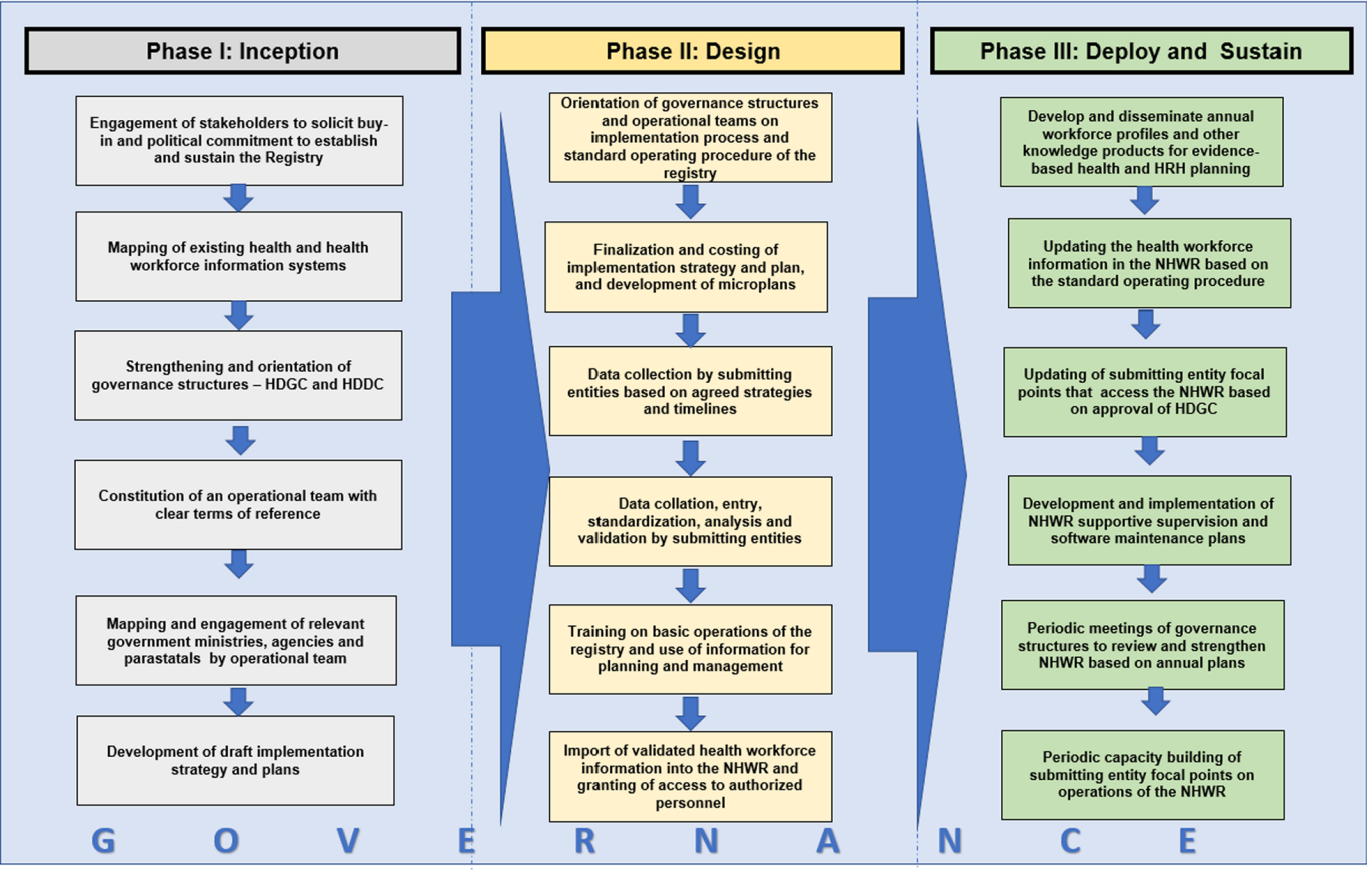
Conceptual framework of the NHWR

The implementation guide provides information on the important steps to take while decentralizing the NHWR. It also proposes processes that enable HDCC, operational teams, and submitting entities across the various levels of care should consider while collecting, collating, and managing, generating accurate health workforce information for health and HRH planning. A three-stage conceptual framework was developed, based on the learnings of the pilot, to guide the establishment, operationalization, decentralization, and sustainability of NHWR (Fig. 1). The framework has three (3) main domains; inception, design, and deploy and sustain, which are all led by governance structures. The inception phase consists of two key activities—soliciting buy-in and establishing or strengthening governance structures. These activities focus on utilizing building political commitment for the establishment of the registry as well as oversight by governance structures in the processes of developing and sustaining the registry. In the design phase, the microplanning step focuses on applying a microplanning template to map the health workers by departments, agencies, and parastatals, local government area, ward, and service delivery points. The completed microplan provides detailed data on the total stock of HRH and is useful in planning for the data collection process. The data collection plan is developed based on the contextual routine data flow processes and implementation. Afterwards, data management supervised by the HDCC and operational team ensues with confidentiality of the health workforce information ensured. The standardized health workforce information is validated and imported into the NHWR with annual health workforce profiles developed and availed to policymakers and health managers for evidence-based planning. In the final deploy and sustain phase, the capacity of relevant stakeholders is periodically built on how to use, manage and administer health workforce information in the registry. This is done with aid of the basic user training manual with hands-on sessions conducted using the NHWR demo and live sites. With the health sector being dynamic with recruitments, promotions, redeployments, and exits, periodic updating through a bottom-top process is essential for the registry to have current information. Sustaining the functionality of the Registry focuses on bottom-top processes of updating the health workforce information in the NHWR following the guidance in the standard operating procedure.

The standard operating procedure provides a guide for data management, use, privacy, security, confidentiality, and frequency of updating and reporting on the registry. It describes the data sets and elements of the NHWR, provides guidance on the data collection, aggregation, and standardization processes, defines the data flow process from submitting entities to the NHWR, and proposes data use options.

Health workforce information from the departments, agencies, and parastatals, and the private sector service providers are submitted by the HRH division/branch/unit at the Ministries of Health. Information from the training institutions (both public and private) and the sub-national regulatory councils are submitted to the professional regulatory bodies. The Ministry of Health, and the professional regulatory bodies, with technical assistance from the operational team updates the information on health workers in the NHWR periodically. The authenticated and validated health workforce information are collated and centrally stored in the NHWR using a bottom-top process. Reports generated from the NHWR are published annually as country health workforce profiles and other knowledge management products to guide decision-makers and policy formulators in managing and planning for the health sector and HRH at all levels.

The basic user manual provides information in executing basic functions on the registry’s online platform progressively. Guidelines for executing the registry’s administrative functions, contingencies, and alternate modes of operation were provided in the advanced user manual. The disaster recovery plan describes the backup and recovery environment for the NHWR should an unintended incident or disaster occurs.

### HRH information

Data on the HRH for 11 states were successfully imported into the NHWR [23]. These states are distributed across five of the six geopolitical regions in the country, with the North–East region having the highest with four states; Adamawa, Bauchi, Borno, and Yobe; while the North-Central, North–West, and South–South zones had one state each; Niger, Sokoto, and Osun, respectively. The South–East and South–West regions had two states apiece with Anambra and Abia as well as Cross River and Edo states.

Overall, a total of 89,988 health worker records were imported into the NHWR. These stock were drawn from 50 submitting entities across these states, with states in the North–East regions accounting for about 50% of the total number. North–West and South East both accounted for 12% each, North Central 10%, while South West and South South had 8%.

**Fig. 2:**
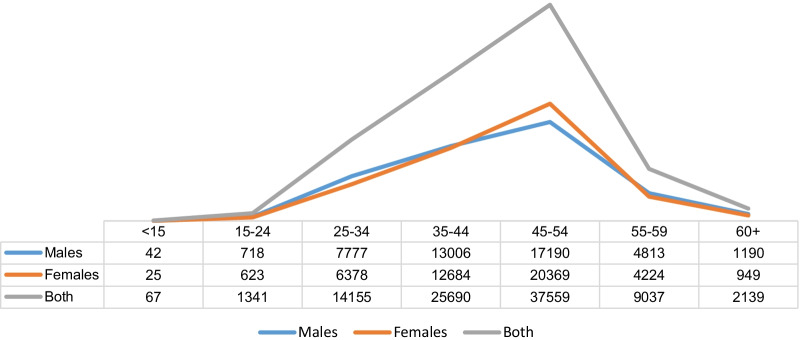
Age distribution of health workforce data from 11 states

Overall, about 97% of the total workforce were within 15–59 years: 15–24 years—1%; 25–34 years—25%, 35–44 years—39%; 45–54 years—42%, and 55–59 years—10%. The highest proportion (42–38% males and 45% females) of the health workforce across the 11 States were within 45–54 year age group (Fig. 2).

**Fig. 3:**
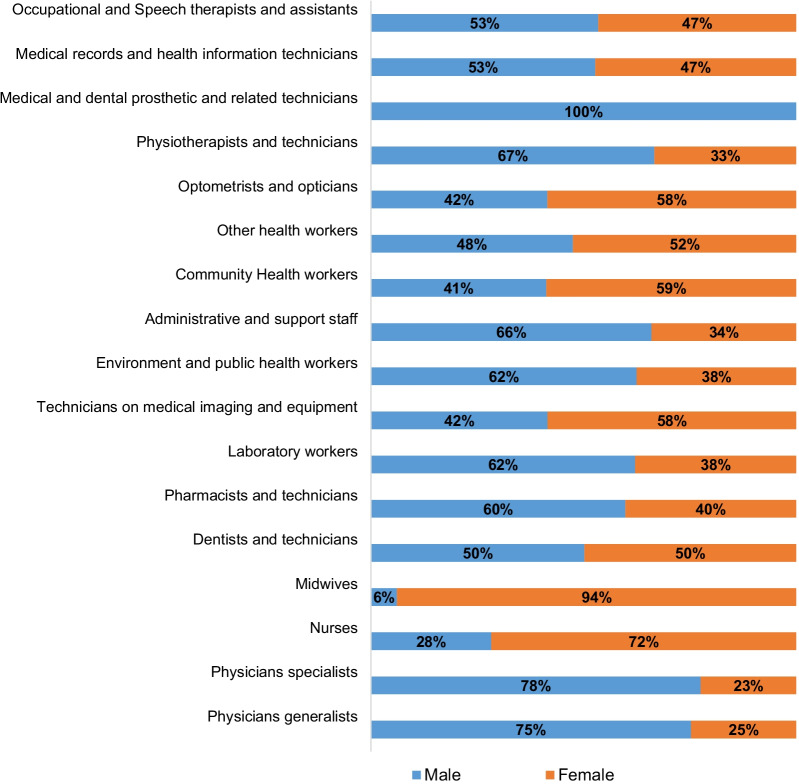
Gender distribution of health worker groups of 11 states

Applying the ISCO classification [24], other health workers outside those presented in Fig. 3 had the highest number (30,551) and proportion (34%). They are followed by 18,233 community health workers (community health officers, community health extension workers and junior community health extension workers) representing 20% and administrative and support staff (16,384 representing 18%). Eleven percent of the cadres (9332) were nurses and midwives, and 2% (1510) were general and specialist physicians. Figure 3 shows the gender distribution of the health worker groups with 35% of the groups being female-dominated.

## Discussion

This case presentation describes the steps taken in developing, decentralizing, and ensuring the sustainability of the NHWR. The findings from the consultations shaped the approach taken to establish a sustainable and scalable NHWR and they were in alignment with the literature [25–27]. Key amongst them was the duplicity of various systems that were not interoperable with the national health information systems and the lack of guidelines and standardized tools for the NHWR. Others were weak governance mechanisms for health workforce information and low capacity amongst relevant stakeholders as well as the absence of capacity building materials for training and mentoring HRH managers saddled with the responsibility of managing the NHWR.

To address these, our approach applied several strategies. They include ensuring that NHWR is interoperable with DHIS, data were collected from multiple sources and triangulated, data sets and elements are standardized and relevant for health and HRH planning and management, capacity building and sustainability guides were developed and a mechanism is in place for use of HRH data for evidence-based policy, strategy and guidelines development [25–27].

The establishment of strong governance structures comprising of policymakers, health managers, and implementers to lead advocacy, dialogues, coordination, and planning is associated with the improvement and sustenance of information systems [28, 29]. For the NHWR, incorporation of this function into the existing and decentralized health information system governance structures was a step that would ensure sustainability. In addition, through consultations and dialogues at various levels, the views and involvement of various stakeholders at several levels shaped and improved the development and decentralization of the NHWR [4, 28, 30]. We recommend that this approach be sustained as the registry is rolled out to other submitting entities in the country and maintained.

The existence of a registry without its application and use is not beneficial to the health system [28, 31, 32]. To ensure this, the Handbook of the National Health Workforce Registry that contains an implementation guide, a standard operating procedure, and a basic user manual [13], the advanced user manual, and the disaster recovery plans were developed and used for capacity building sessions at various levels. This served to deepen the understanding of the governance structures, operational team and HRH focal persons at national and sub-national levels on the programmatic and software operations of the NHWR. They also serve as a reference guide for users of the system and inform future capacity-building activities as the registry is decentralized and used [4, 33]. Ultimately, this would sustain the NHWR.

The NHWR has great potentials and its appropriate use will end the prolonged absence of a reliable source for accurate and quality HRH data for the health sector and HRH policy, planning, and management. In the context of HRH strengthening, the information generated from the registry will provide the basis for formulating policies needed to shape Nigeria’s health labor market towards achieving national health sector and HRH goals, UHC, and the SDGs. Specifically, policies to improve the production of appropriately skilled health workers with the right skill-mix by cadres, address health workforce attraction and migration, improve skill-mix for service delivery and address inequitable distribution, and enhance regulation of service providers (public and private) and performance management[1, 24, 34]. In some countries, information from the registry has been useful in National Health Workforce Accounts reporting and developing key documents including health sector strategic plans, HRH strategic plans, annual HRH Country Profiles, HRH production plans, HRH deployment plans, staffing needs assessments, at national and sub-national levels, and national HRH Production Plans [11, 24, 35, 36].

The unavailability of complete and contemporary health workforce information remains a big challenge in several countries despite its importance in planning towards achieving national goals and UHC [37–39]. This is often linked to several factors including the way country-level health workforce registries are conceptualized and designed, implemented, and governed, decentralized amongst other sub-national levels as well as sustained [40, 41]. This study contributes to the body of knowledge on strategies for conceptualizing, designing and implementing a sustainable and scalable health workforce registry and this process is replicable in any resource-constrained environment. In addition, this study considers the complexity of the health system in the decentralization process and the highlights strategies for ensuing sustainability.

We faced some challenges in the course of conceptualizing and implementing the registry. While reviewing previous endeavors towards establishing registries, some stakeholders were very reluctant to share complete information on the content and functionality of exiting systems. In addition, due to varying interests to meet project obligations to donors, the approach to have a holistic system aligned to the existing health management information system architecture was resisted by some stakeholders. This was also experienced during the data tool development process during which implementers of various global health initiative programs tried to skew the registry to meet their program needs rather than the broader health system’s needs. In addition, due to the sensitivity of collecting health workforce details, there were challenges experienced during the data collection and management phase. The aforementioned challenges were mitigated by applying several strategies including having a multi-stakeholder team that were driven by a systems strengthening approach to deliver a holistic registry, ensuring transparency in the implementation process and the strong political will of the leadership of the FMoH.

We learned some key lessons in this process. In establishing a functional and sustainable HRH registry, learning from previous experiences is essential in shaping acceptable, sustainable, and scalable approaches. Instituting governance structures that include and involve policymakers, health managers and users is of great importance in the design, planning, implementation, and decentralization stages. In addition, developing standardized tools based on the health system's needs and instituting supportable mechanisms for data flow and use for policy, planning, development, and management is essential. In this instance, developing the national guidelines for future reference was a key milestone.

## Conclusions

Ensuring that the right number of health workers that are qualified, skilled, and distributed equitably are available for quality health service provision requires accurate and timely health workforce information. Conceptualizing and establishing health workforce registries should be informed by contextual needs and strategies to ensure sustainability.
